# Dupuytren’s interventions surgery versus collagenase (DISC) trial: study protocol for a pragmatic, two-arm parallel-group, non-inferiority randomised controlled trial

**DOI:** 10.1186/s13063-021-05595-w

**Published:** 2021-09-30

**Authors:** Joseph Dias, Catherine Arundel, Puvan Tharmanathan, Ada Keding, Charlie Welch, Belen Corbacho, Maria Armaou, Paul Leighton, Christopher Bainbridge, Michael Craigen, Lydia Flett, Samantha Gascoyne, Catherine Hewitt, Elaine James, Sophie James, Nick Johnson, Judy Jones, Catherine Knowlson, Priya Radia, David Torgerson, David Warwick, Michelle Watson

**Affiliations:** 1 University Hospitals of Leicester NHS Trust, Leicester, UK; 2 York Trials Unit, University of York, York, UK; 3 University of Nottingham, Nottingham, UK; 4 University Hospitals of Derby and Burton NHS Trust, Derby, UK; 5 The Royal Orthopaedic Hospital, Birmingham, UK; 6 University Hospital Southampton, Southampton, UK

**Keywords:** Dupuytren’s contracture, Collagenase clostridium histolyticum, Limited fasciectomy, Surgery, Correction, Randomised controlled trial

## Abstract

**Background:**

Dupuytren’s contracture is a fibro-proliferative disease of the hands affecting over 2 million UK adults, particularly the white, male population. Surgery is the traditional treatment; however, recent studies have indicated that an alternative to surgery—collagenase clostridium histolyticum (collagenase)—is better than a placebo in the treatment of Dupuytren’s contracture. There is however no robust randomised controlled trial that provides a definitive answer on the clinical effectiveness of collagenase compared with limited fasciectomy surgery. Dupuytren’s intervention surgery vs collagenase trial (DISC) trial was therefore designed to fill this evidence gap.

**Methods/design:**

The DISC trial is a multi-centre pragmatic two-arm parallel-group, randomised controlled trial. Participants will be assigned 1:1 to receive either collagenase injection or surgery (limited fasciectomy).

We aim to recruit 710 adult participants with Dupuytren’s contracture. Potential participants will be identified in primary and secondary care, screened by a delegated clinician and if eligible and consenting, baseline data will be collected and randomisation completed.

The primary outcome will be the self-reported patient evaluation measure assessed 1 year after treatment. Secondary outcome measures include the Unité Rhumatologique des Affections de la Main Scale, the Michigan Hand Questionnaire, EQ-5D-5L, resource use, further procedures, complications, recurrence, total active movement and extension deficit, and time to return to function. Given the limited evidence comparing recurrence rates following collagenase injection and limited fasciectomy, and the importance of a return to function as soon as possible for patients, the associated measures for each will be prioritised to allow treatment effectiveness in the context of these key elements to be assessed.

An economic evaluation will assess the cost-effectiveness of treatments, and a qualitative sub-study will assess participants’ experiences and preferences of the treatments.

**Discussion:**

The DISC trial is the first randomised controlled trial, to our knowledge, to investigate the clinical and cost-effectiveness of collagenase compared to limited fasciectomy surgery for patients with Dupuytren’s contracture.

**Trial registration:**

Clinical.Trials.gov ISRCTN18254597. Registered on April 11, 2017.

**Supplementary Information:**

The online version contains supplementary material available at 10.1186/s13063-021-05595-w.

## Administrative information

The order of the items has been modified to group similar items (see http://www.equator-network.org/reporting-guidelines/spirit-2013-statement-defining-standard-protocol-items-for-clinical-trials/).

**Table:** 

Title {1}	Dupuytren’s interventions surgery versus collagenase (DISC) trial: study protocol for a pragmatic, two-arm parallel-group, non-inferiority randomised controlled trial
Trial registration {2a and 2b}.	ISRCTN18254597. Prospectively registered on 11th April 2017 EudraCT 2016-004251-76. Prospectively registered on 21st October 2016 Retention Thank You Card SWAT - MRC Hub for Trials Methodology Research SWAT repository #119 Registered 13.04.2020 Retention Christmas Card SWAT - MRC Hub for Trials Methodology Research SWAT repository #82 Registered 24.08.2017
Protocol version {3}	Version 2.2 28.07.2020
Funding {4}	This project was funded by the National Institute for Health Research Health Technology Assessment (project number 15/102/04). The views and opinions expressed therein are those of the authors and do not necessarily reflect those of the Health Technology Assessment Programme, NIHR, NHS or the Department of Health.
Author details {5a}	Please see the cover page for the full list of DISC trial Protocol Authors
Name and contact information for the trial sponsor {5b}	University Hospitals of Leicester NHS Trust, Trust HQ, Level 3 Balmoral Building, Leicester Royal Infirmary, Infirmary Square, Leicester LE1 5WW
Role of sponsor {5c}	Study sponsor and funder have had no role in study design nor in the collection, management, analysis or interpretation of data. They will have no role in the writing of associated publications and the decision to submit papers for publication.

## Introduction

### Background and rationale {6a}

Dupuytren’s contracture is a fibro-proliferative disease, common in the White, male population, affecting over two million UK adults. The contracture forms nodules and cords at the metacarpophalangeal (MCP) joint and/or proximal interphalangeal (PIP) joint, drawing down the finger into a flexed position. This results in an inability to straighten the finger, which impacts on a patient’s quality of life.

Surgical correction of the contracture, by dissecting the cords and removing them (limited fasciectomy), is the standard treatment in the UK and Europe [1, 2]. Over 17,000 of such operations occur in England each year, resulting in costs to the NHS of more than £60 million per annum [3]. Patients may experience infection, delayed healing, nerve damage, pain, stiffness and recurrence in addition to more serious complications (e.g. vascular compromise and complex regional pain syndrome) which may delay recovery and impair function.

An alternative approved treatment for Dupuytren’s contracture is the dissolution of the cord by injecting an enzyme, collagenase *clostridium histolyticum* (collagenase), followed by manually snapping the weakened cord to correct the contracture. Collagenase was registered for use in the USA in 2010 and continues to be used frequently. European registration was completed in 2011; however, deregistration in Europe occurred in March 2020 due to commercial, rather than safety or efficacy, considerations [4]. Patient recovery may be delayed while short-term side effects (e.g. swelling, pain, skin splits) resolve and no long-term systemic effects have been identified. Tendon rupture however is a significant but uncommon complication which may require surgical intervention [5]. Patients treated may recover function and undertake routine tasks more quickly with collagenase injection than with surgery [4–8].

A further potential benefit of collagenase injection, over limited fasciectomy, is that the procedure can be conducted within a clinic setting by a variety of trained clinicians. Although patients do need to attend additional clinic visits to complete the procedure, they do not need to wait for and subsequently undergo surgery, thus releasing surgical capacity within the healthcare setting.

Initial clinical effectiveness studies comparing collagenase with placebo [5] and a systematic review in 2015 [9] indicated that collagenase is better than placebo, particularly for contractures affecting the MCP joint [5]. Observational data on recurrence of Dupuytren’s contracture (a change in extension deficit of 6° between 3 and 6 months, or 20° from 3 months to 1 year post-treatment [6, 10]) suggest that it is higher following collagenase treatment than surgery 3 years after treatment.

Current evidence of the cost-effectiveness of collagenase and surgical correction is based on small retrospective studies [9] and so conclusions are limited.

Despite the existing available evidence, there is no robust randomised controlled trial evidence available on the clinical and cost-effectiveness of collagenase compared with limited fasciectomy surgery. There is also currently no robust evidence on the comparative recurrence rates of Dupuytren’s contracture following treatment with collagenase compared with limited fasciectomy surgery. A sufficiently powered randomised controlled trial is required to fill this evidence gap. Therefore, the UK National Institute for Health and Care Excellence (NICE) commissioned a full-scale RCT to provide a definitive answer on the effectiveness of these treatment options.

Overall, collagenase injections would have to not be inferior to limited fasciectomy surgery to permit recommendation for general use. The Dupuytren’s Interventions Surgery vs Collagenase (DISC) trial was designed as a pragmatic multi-centre, randomised controlled, non-inferiority, cost-effectiveness trial comparing injections of collagenase into the cord to surgical correction in the treatment of moderate Dupuytren’s contracture in adult patients.

### Objectives {7}

The objectives of the trial are listed in Table 1.

### Trial design {8}

The DISC trial is a two-arm, pragmatic, non-inferiority, randomised controlled trial investigating whether collagenase injection is not inferior to limited fasciectomy in the correction of Dupuytren’s contracture of the hand. The trial includes an economic evaluation, a qualitative sub-study, a photography sub-study and two nested studies within a trial of retention strategies.

**Table 1:** DISC trial objectives

Objective	
1	To investigate whether collagenase injection is not inferior to limited fasciectomy in the *(impact of)* correction of Dupuytren’s contracture of the hand at 1 year^a^.
2	To investigate whether the *(impact of)* correction achieved after collagenase injection or surgical correction is maintained at 1 and 2 years^a^.
3	To investigate the cost-effectiveness of collagenase injections compared to limited fasciectomy at 1 and 2 years after treatment (from the NHS and Personal Social Services perspectives).
4	To investigate if the remote measurement of active extension and flexion using photographs is as good as goniometric measurements in a clinic to determine recurrence (photography sub-study).
5	To explore patient’s experiences and preferences of the different treatments (qualitative sub-study).

^a^*In terms of patient-reported hand pain and functionality as assessed by the PEM, as further detailed in the “Outcomes” section*

## Methods: Participants, interventions and outcomes

### Study setting {9}

The DISC trial is recruiting from 31 secondary care hand units in the NHS hospital trusts in the UK. Identification of study sites will be supported by the British Society for Surgery of the Hand (BSSH). A list of study sites is provided as Supplementary File 1.

### Eligibility criteria {10}

The trial will include adult patients with Dupuytren’s contracture who meet all of the inclusion criteria, and none of the exclusion criteria.

#### Inclusion

- Male or female and aged 18 years or over.
- Presence of discrete, palpable, contracted cord involving the metacarpophalangeal joint and/or proximal interphalangeal joint of a finger.
- Degree of contracture ≥30° in either joint i.e. patient cannot put the palm of the hand flat on a table (Hueston’s tabletop test).
- Able to identify a predominant cord for treatment, which would not require more than one collagenase injection as treatment.
- Appropriate for limited fasciectomy surgery and collagenase injection for Dupuytren’s contracture (i.e. cords suitable for collagenase injection and limited fasciectomy and not requiring skin grafting or percutaneous needle fasciotomy (e.g. discrete metacarpophalangeal joint cords in elderly)).
- The patient is willing and able to give informed consent for participation in the study.

#### Exclusion

- Severe contractures of the metacarpophalangeal joint and/or proximal interphalangeal joint (Tubiana grade 4—total extension deficit greater than 135°).
- History of previous treatment for Dupuytren’s contracture (e.g. surgery, collagenase injection or needle fasciectomy) to the study reference digit.
- History of any other pre-existing disorder of the hand causing significant restriction of movement and/or pain and affecting hand function e.g. post-traumatic stiffness, stiffness due to other causes, infection, arthritis.
- Non-English speaking because of the need to complete multiple questionnaires which have not been validated in multiple languages.
- Resident in a location where attendance for follow-up at one of the studies recruiting centres will not be possible.
- Contraindicated for use of collagenase including:
  - Hypersensitivity to collagenase, sucrose, ketorolac trometamol, hydrochloric acid, calcium chloride dehydrate and sodium chloride
- Diagnosis of a coagulation disorder
- Any other significant disease or disorder (including autoimmune disorders) which, in the opinion of the Investigator, may put the participant at risk because of participation in the study, or may influence the result of the study, or the participant’s ability to participate in the study.
- Participation in another research study involving an investigational product in the past 12 weeks.
- Female participants who report to be pregnant or breastfeeding.

### Who will take informed consent? {26a}

Potential participants will be identified through clinician referrals, surgery and clinic lists, allied clinics and centres (e.g. musculoskeletal and physiotherapy clinics, musculoskeletal triage centres), private practice and GP settings. Eligibility will be assessed and confirmed at the recruiting hospital by a delegated clinician.

Patients will be provided with an information sheet and will be given the opportunity to ask questions about the study. Informed consent will then be obtained by a suitably qualified, experienced and delegated research nurse or clinician. Separate consent forms will be completed for the photography and qualitative sub-studies.

Following a recruitment pause as a result of the COVID-19 pandemic, clinic visits can be completed by video appointment. Where a video appointment is completed for baseline, the consent form must be personally signed by the participant and the delegated clinician prior to treatment delivery. Patients will be provided with guidance on using the relevant software prior to the appointment.

### Additional consent provisions for collection and use of participant data and biological specimens {26b}

There are no biological specimens collected within the DISC trial; therefore, additional consent for the collection and use is not required.

Separate consent will be completed for data collection and use relating to the photography and qualitative sub-studies included within the DISC trial.

### Interventions

#### Explanation for the choice of comparators {6b}

Collagenase injection (intervention) and limited fasciectomy (control) were identified as comparators by the UK National Institute of Health Research Health Technology Assessment Programme as part of a commissioned call (15/102) to assess collagenase as a treatment for Dupuytren’s contracture.

The justification for the choice of these two comparators was that the effectiveness of collagenase compared to placebo had been investigated in randomised controlled trials [5, 9]; however, its performance compared to established treatments had not been rigorously tested.

Limited fasciectomy surgery is the standard treatment for Dupuytren’s contracture in the UK and Europe and hence was selected as the comparator.

#### Intervention description {11a}

Patients will be scheduled for intervention (collagenase injection) or control (limited fasciectomy surgery) treatment within 18 weeks of randomisation (as per recommended referral to treatment time (RTT)); however, sites will be asked, where possible, to complete the treatment procedure within 12 weeks of randomisation.

Separate cords can be injected (intervention) or operated on (control) at the same treatment visit; however, a reference cord (i.e. predominant) must be identified prior to randomisation, with follow-up assessments (e.g. recurrence, further procedures etc.) being based primarily on this cord.

#### Collagenase clostridium histolyticum (intervention)

Collagenase clostridium histolyticum (Xiapex) is an enzyme activated by mixing a powder with fluid in set quantities (0.58mg) immediately prior to injection.

When the DISC trial commenced, collagenase was manufactured by Auxilium and marketed by Sobi, Sweden. However, marketing authorisation for collagenase use within Europe was withdrawn in March 2020 by the parent company (Endo) for commercial reasons. The DISC trial has worked extensively with Sobi to facilitate the availability of sufficient vials to enable completion of the study.

Either 0.25ml (MCP joint) or 0.20ml (PIP joint) of reconstituted solution (0.58mg collagenase clostridium histolyticum) will be injected as three aliquots at set anatomical points. After an interval of one to 7 days, the participant will return to clinic and, under local anaesthetic, and the cord will be snapped correcting the contracture.

#### Limited fasciectomy (control)

Limited fasciectomy surgery is where, under anaesthesia, the diseased fascia, nodule and cord, or a part of it, are removed to correct the joint contracture [11, 12]. Following limited fasciectomy, participants will be reviewed at a routine wound check.

#### Criteria for discontinuing or modifying allocated interventions {11b}

Given the nature of the study interventions, it will not be possible to discontinue either intervention once treatment has been delivered.

Collagenase injection will be delivered in accordance with the current approved summary of product characteristics for collagenase clostridium histolyticum [4]. With approval from the UK competent authority (Medicines and Healthcare products Regulatory Authority - MHRA), the interval for joint manipulation following collagenase injection will be 1 to 7 days.

#### Strategies to improve adherence to interventions {11c}

Given the nature of the study interventions, no specific strategies have been included to improve intervention adherence.

#### Relevant concomitant care permitted or prohibited during the trial {11d}

Additional clinical review, further treatments, including collagenase injections, and concomitant medications will be determined by clinical need and will be recorded in follow-up case report forms (CRFs).

#### Provisions for post-trial care {30}

At the end of the trial, participants will return to the care of their treating healthcare professional to determine any further treatment as required.

#### Outcomes {12}

##### Main study

Table 2 outlines the time points where outcomes are assessed, with further details regarding these provided below.

**Table 2:** Outcome measure assessment time points

Procedures	Baseline	Treatment delivery	Week 2 post-treatment	Week 6 post-treatment	3 months post-treatment	6 months post-treatment	1 years post-treatment	2 years post-treatment
Informed consent	**x**							
Demographics	**x**							
Condition history	**x**							
Compliance					**x**			
Joint measurements (goniometry) for extension and total active movement	**x**	**x**						
Joint measurements (goniometry) for extension deficit, recurrence and total active movement					**x**	**x**	**x**	**x**
Diathesis indicators	**x**							
Comorbidity index	**x**							
Clinical assessment of cords	**x**							
Randomisation	**x**							
Intervention/control procedure scheduled	**x**							
Treatment delivered		**x**						
Concomitant medications	**x**				**x**	**x**	**x**	**x**
Photographs of the hand	**x**	**x**			**x**	**x**	**x**	**x**
Patient evaluation measure (PEM)	**x**	**x**			**x**	**x**	**x**	**x**
Unité Rhumatologique des Affections de la Main (URAM) Scale	**x**				**x**	**x**	**x**	**x**
Michigan Hand Questionnaire	**x**						**x**	**x**
EQ-5D-5L	**x**		**x**	**x**	**x**	**x**	**x**	**x**
Further procedures and complications					**x**	**x**	**x**	**x**
Resource use					**x**	**x**	**x**	**x**
Adverse event assessments					**x**	**x**	**x**	**x**
Remote collection of patient procedure experience			**x**	**x**				

##### Primary outcome

The primary outcome will be the score for part two of the patient evaluation measure (PEM) [13] at 1-year post-treatment.

The PEM is a validated patient report questionnaire, which includes 11 items relating to hand health and three items relating to overall assessment (including a transition question). PEM was chosen as the primary endpoint due to its ability to capture changes in patient’s hand health after treatment as compared to other validated measurement tools for the hand, which have been included as secondary outcomes [14].

##### Secondary outcomes

Secondary outcomes include patient-reported and clinical outcome measures. Given the limited available evidence comparing recurrence rates following collagenase injection and limited fasciectomy, and as patient representatives have noted the importance of a return to function as soon as possible following treatment, relevant measures for each will be included and prioritised to allow treatment effectiveness in the context of these key elements to be assessed.

##### URAM patient-rated outcome measure

The Unité Rhumatologique des Affections de la Main (URAM) is a validated, nine item, six interval disease-specific disability scale [15].

##### Michigan Hand Questionnaire

The Michigan Hand Questionnaire (MHQ) is a validated, 63-question measure assessing each hand individually via six domains: overall hand function, activities of daily living, work performance, pain, aesthetics and patient satisfaction with hand function [16, 17]. The function and pain domains refer to patient symptoms whilst work and activities of daily living refer to disability and handicap.

##### Objective measures (recurrence, extension deficit and total active movement)

Recurrence (defined as a change in extension deficit of 6° between 3 and 6 months, or 20° from 3 months to 1 year after treatment [6, 10]), extension deficit and total active movement will be assessed using joint range measurements with a goniometer. A study-specific manual on how to perform joint measurements and use of goniometers which meet pre-specified criteria (permits measurement of up to 30° of hyperextension, measures flexion to 120°, measures in 2° increments) will ensure assessments are standardised.

Photographs of the participant’s reference hand (extension, flexion and AP views) will be taken in clinic. A study-specific manual will be used to standardise the images. If willing, participants will also take and return a photograph of their hand, again standardised using a study-specific procedure and video provided to participants

##### Further procedures

Information on further procedures or treatment required for the participant’s Dupuytren’s contracture will be collected at each follow-up assessment.

##### Complications

Complications relating to the intervention and control treatments will be recorded. Expected complications for the intervention arm are listed in the Summary of Product Characteristics for Collagenase Clostridium Histolyticum [4]. Expected complications for the control (limited fasciectomy) arm are listed in Table 3.

##### EuroQol (EQ-5D-5L)

The EQ-5D-5L [18] is a validated, generic, patient-reported health status measure. This assesses five dimensions of health (mobility, self-care, usual activities, pain/discomfort and anxiety/depression) on five severity levels (no problems, slight problems, moderate problems, severe problems, and unable/extreme problems). A visual analogue scale (VAS) from 100 (best imaginable health) to 0 (worst imaginable health) [18], also records participant’s overall evaluation of their health. The EQ-5D-5L will allow us to assess health-related quality of life outcomes in the economic evaluation.

**Table 3:** Expected complications associated with limited fasciectomy surgery

Amputation	Scar pain
Arterial injury	Scar related complications (including hypertrophy)
Bleeding	Stiffness
Complex Regional Pain Syndrome (CRPS)	Swelling
Delayed healing	Tendon injury
Infection	Edge necrosis
Instability	Carpal tunnel syndrome (starting within six weeks of limited fasciectomy)
Nerve injury	Other – Tenosynovitis (starting within 6 weeks of limited fasciectomy)
Pain	Other - Trigger finger (starting within 6 weeks of limited fasciectomy)
Paraesthesia (including dysaesthesia, burning and hyperaesthesia)	

##### Resource use

Forms specifically designed for the DISC trial will collect resource use from hospital records and through participant self-report. This will include health resource use (treatment delivery, inpatient episodes, outpatient visits, emergency hospital admissions and primary care visits (e.g. GP, nurse and physiotherapy)) in addition to return to work and out-of-pocket expenses.

##### Time to recovery of function

Time to recovery of function will be assessed using a Single Assessment Numeric Evaluation (SANE) measure [19]; a single question, a patient-reported measure which assesses patient functionality.

##### Qualitative sub-study

Approximately 40 participants, selected purposively from those who consent to this sub-study, will take part in a semi-structured interview to generate data on the benefits and difficulties that patients perceive with each treatment. Initial recruitment will target ‘typical cases’ (where there are no complications) with subsequent recruitment informed by emergent issues (in the interview data or main trial conduct). We expect to recruit similar numbers from each arm of the trial.

Interviews will coincide with outcome data collection at 3 months. Questions and topics will include: Dupuytren’s symptoms and impact upon lifestyle, expectations and experience of treatment, recovery and progress in everyday activities, future concerns, treatment preference; and recommendations for clinical guidelines. Interviewees will also be given the opportunity to raise any other issues which they consider pertinent.

##### Photography sub-study

To give the widest range of angles possible, we will recruit sufficient participants to provide 100 photograph sets before surgery and will also collect as many photograph sets as possible at 3 months, 6 months, 1 year, and 2 years post-treatment.

Participants who consent to participate in the photography sub-study will be shown how to take the required photographs of their hand at baseline and will be provided with detailed instructions. This will include a template to aid placement of the participant’s hand, an information leaflet on how to submit photos, and a link to a video showing how to take photographs. Documentation will be reviewed and approved by the patient and public involvement group prior to use in the study.

Sub-study participants will be asked to take standardised photographs of their study reference hand at baseline and all subsequent time points. Participants will receive a text message or letter at each time point to remind them to return photographs to the study team for this sub-study via email, SMS or post.

##### Nested retention studies within a trial (SWAT)

We will undertake two nested randomised controlled trials. One will evaluate the effectiveness of a thank you card on participant retention; the other the effectiveness of a festive greetings card on participant retention. The inclusion of these SWATs was not planned at the outset of the trial, and therefore, due to the timing of identification and implementation (thank you cards decision December 2018, decision July 2019, implementation May 2019; festive greetings card implemented December 2019), it will not be possible to utilise a factorial design and so each will be handled as a separate SWAT.

In the first SWAT, participants will be allocated to either receive a thank you card at 9 months post-randomisation or to not receive a thank you card. In the second SWAT, participants will be allocated to receive a festive greetings card or to not receive a card.

All participants randomised into the main DISC Trial and who remain as fully participating (i.e. have not fully withdrawn, withdrawn from postal follow-up or have died) will be eligible and randomised for each of the two SWATs. The exception will be DISC participants due to receive a thank you card in December 2019 who will be excluded from the festive greeting card SWAT.

The primary outcome of both embedded trials will be the difference in retention rate between those who receive the card, and those who do not. The cost per participant retained will serve as a secondary outcome for both SWATs. The thank you card SWAT will also assess the completeness of outcome data, and the Christmas card SWAT will assess time since intervention and response rate.

#### Participant timeline {13} (Fig. 1)

##### Sample size {14}

Previous survey data (880 patients) showed the standard deviation of the scores for the 11 items in part 2 of the primary outcome (PEM) at baseline to be 22 points (unpublished). We estimate that a six-point difference on the PEM at 12 months is the threshold at which treatment differences become important and represents an appropriate non-inferiority margin.

**Fig. 1:**
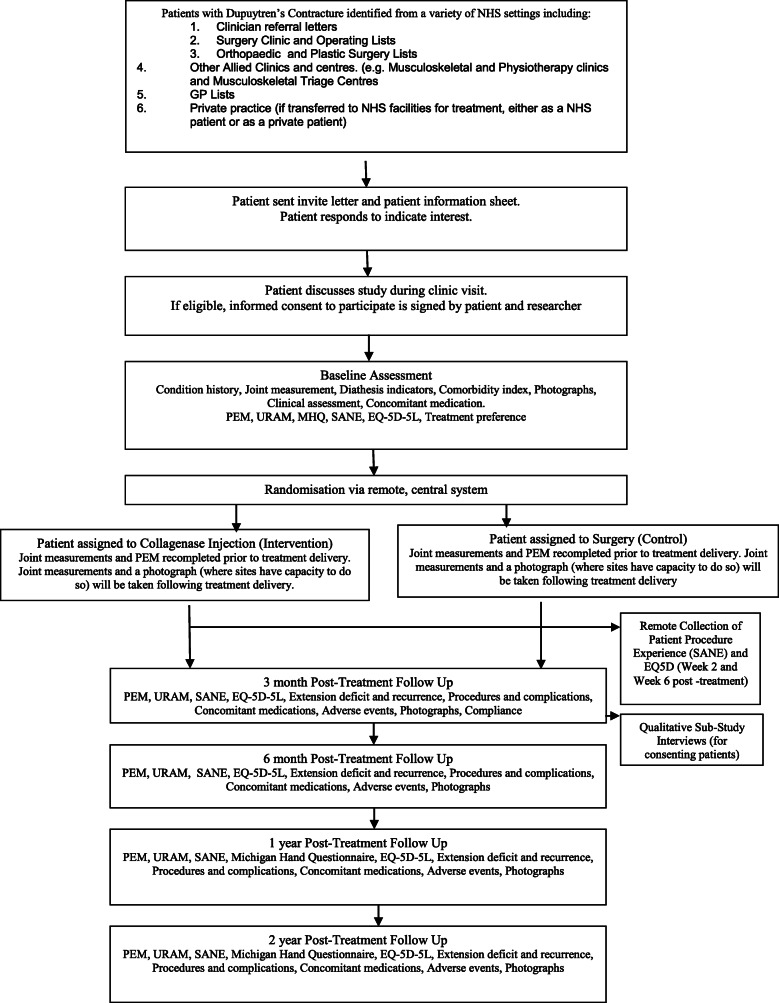
Participant flow diagram

An effective sample size of 568 participants (284 per arm) is required to obtain 90% power to assess non-inferiority of collagenase based on the upper limit of a two-sided 95% confidence interval (equivalent to a one-sided 97.5% CI) for the treatment difference (collagenase - surgery) at 1-year post-treatment, assuming a non-inferiority margin of six points and a standard deviation of 22 points. Assuming 20% attrition at the 1-year follow-up, the total target sample size is 710. The effective sample size of 568 is based on a (one-sided) independent samples *t* test of size 2.5%, neglecting information provided by informative baseline covariates (reference joint and baseline PEM score) and repeated measurements of the PEM.

The primary analysis will condition on these baseline covariates and will model PEM scores at all post-treatment time points using a covariance pattern model. Assuming the model assumptions are at least approximately satisfied, this approach would be expected to deliver greater statistical power at a given sample size, than the unconditional analysis on which the chosen sample size is based.

##### Recruitment {15}

Strategies for achieving adequate participant recruitment will include seeking advice from our patient focus group and completion of recruitment evaluation interviews with site teams.

Trial training and discussions in relation to key study elements will be implemented through face-to-face meetings with the PI’s at BSSH conferences and routine site investigator meetings.

Research teams will be provided with training at a site initiation visit and a trial manual will be provided to ensure adherence to trial processes. Support and guidance will be provided to staff when required (e.g. when new staff join or replace existing site staff) with clinical guidance from the Chief Investigator when necessary.

#### Assignment of interventions: allocation

##### Sequence generation {16a}

Eligible, consenting participants will be randomised 1:1 to either collagenase injection or limited fasciectomy. Block randomisation with randomly varying block sizes will be used, stratified by the reference joint type (MCP or PIP).

As a result of the withdrawal of marketing authorisation for the study intervention (collagenase), resulting in limited availability within the UK, the randomisation sequence was amended, with effect from the 21st of January 2020, to include stratification by the centre.

In both SWATs, participants will be randomised in a 1:1 ratio, stratified by DISC trial allocation.

##### Concealment mechanism and Implementation {16b} {16c}

The trial statistician, independent of the participating NHS Trust sites, will generate the allocation sequence for the main trial. Randomisation will be completed using a secure, central online randomisation service hosted by Sealed Envelope Ltd.

Treatment will be allocated on an individual named participant basis, and the participant will receive their allocated treatment as soon as possible after randomisation. Following randomisation, a letter will be sent to the GP for filing in the participant’s records.

The trial statistician will also generate the allocation sequence for the retention SWATs and will provide the randomised allocations to the central coordinating team for implementation.

#### Assignment of interventions: Blinding

##### Who will be blinded and procedure for unblinding if needed {17a/17b}

Neither the study design nor the interventions allow for the masking of clinicians or participants; therefore, an unblinding procedure is not required for this study.

The interventions used in the nested sub-studies are deemed to be low risk, and therefore, unblinding for these components is not required.

Due to the way in which data will be collected will also not be possible to blind the statistician to trial allocation for the analysis. All analyses will be pre-specified in a statistical analysis plan, and any changes made to the data set prior to analysis will be documented appropriately.

#### Data collection and management

##### Plans for assessment and collection of outcomes {18a}

Case report forms (CRFs) will record all required information with separate CRFs collecting clinical and patient-reported information. Copies of CRFs used in the trial are available on request.

##### Plans to promote participant retention and complete follow-up {18b}

Participants will receive £40 following completion of each of the 1-year and 2-year participant outcome questionnaires. This strategy has been found to have an effect on improving participant retention and questionnaire response rates [20, 21].

Participants will also be sent a study newsletter 4 weeks before the 1-year time point to maintain engagement with the trial and to encourage attendance at the 1-year study visit. This will be accompanied by a cover letter from the study Chief Investigator to thank them for their continued contribution to the study.

The coordinating centre at the University of York will liaise with trial sites to ensure that visits for the 1-year primary outcome time point are arranged accordingly. Where a visit cannot be arranged within 4 weeks of the visit due date, a postal questionnaire will be sent to the participant. If there is still no response after a further 4 weeks, the participant will be telephoned to collect their data.

A SWAT testing a thank you card is also embedded within the DISC trial. Participants will be individually randomised to either receive a thank you card at months 9 months post-treatment, or to receive a no-thank-you card at this time point.

##### Data management and confidentiality {19} {27}

All data will be stored and handled in accordance with data protection principles.

Data will be entered, checked and validated using an automated, electronic system (Teleform) as licensed for use at the UKCRC-registered University of York Trials Unit (YTU). A data management plan will document details of the data processing.

All participant data will be identified by a coded number to maintain participant confidentiality.

Paper documents will be stored in locked cabinets in restricted access areas either at the University of York or at an alternative secure off-site facility. Electronic records will be stored on a password-protected server.

Photographs of participant’s hands will be anonymised prior to electronic transfer by sites to YTU. Images will be stored in an encrypted and password-protected drive and will be transferred via secure and encrypted systems to the University Hospitals of Leicester NHS Trust for processing. Participant details required to facilitate contact and arrangement of the qualitative sub-study will be provided to colleagues at the University of Nottingham and University Hospitals of Leicester NHS Trust via secure and encrypted systems.

Participants’ data may be reviewed by authorised persons to verify that the study is being carried out correctly; all of whom will have a duty of confidentiality. Trial participants will give permission for this at the time of consent. All names and other identifying information will be removed before the data are analysed and the results presented and published.

Essential trial documentation relating to the conduct of the trial and the data produced will be kept within the Trial Master File and Investigator Site Files. This documentation will be is retained in accordance with guidelines on Good Research Practice and UK Law. Paper data will be disposed of securely and electronic data will be anonymised.

##### Plans for collection, laboratory evaluation and storage of biological specimens for genetic or molecular analysis in this trial/future use {33}

No plans are required for collection, evaluation or storage biological specimens given; these are not collected within the DISC trial.

## Statistical methods

### Statistical methods for primary and secondary outcomes, additional analyses and handling of non-adherence and missing data {20a, 20b, 20c}

#### Statistical analysis

A detailed analysis plan will be agreed upon with the Data Monitoring and Ethics Committee prior to the end of recruitment. Any subsequent amendments will be clearly stated and justified.

Baseline data will be summarised descriptively by allocation and overall, both as-randomised and as-analysed. The as-randomised set will include all randomised patients, except any ineligible patients randomised in error. The as-analysed set will include all participants with primary outcome data for at least one post-treatment time point. Outcome data will also be summarised descriptively by allocation at all relevant time points. Continuous data will be summarised in terms of the non-missing sample size, mean, standard deviation, median, inter-quartile range and range, and categorical data using frequencies and proportions. The flow of participants through each stage of the trial as well as data completeness will be illustrated in a Consolidated Standards of Reporting Trials (CONSORT) diagram [22].

The primary analysis will include all participants with at least one post-treatment PEM measurement. The between-group differences (collagenase—surgery) in mean PEM score at each post-treatment time point, and two-sided 95% CIs, will be estimated using a covariance pattern model, with all post-treatment PEM measurements included as outcomes. Treatment group and time point and their interaction will be included as fixed effects. The model will also adjust for informative baseline covariates, namely study reference joint and baseline PEM score, as fixed effects and include random intercepts for the study recruitment site. The correlation between repeated measurements will be accounted for using an unstructured covariance matrix. This model will be fitted using restricted maximum likelihood, with degrees of freedom calculated using the Kenward and Roger method [23]. The null hypothesis that collagenase is inferior to surgery will be rejected if the upper bound of the two-sided 95% CI for the estimated difference (collagenase—surgery) in means at 1-year post-treatment exceeds the non-inferiority margin of six points.

Compliance with randomised treatments will be reported, and an instrumental variable estimator (assuming random allocation is a valid instrument) will estimate the Complier Average Causal Effect (i.e. the average causal effect of collagenase compared to surgery among the “complier” principal stratum). The primary analysis assumes missing outcome data are missing at random (MAR) conditional on the baseline covariates and non-missing outcomes included in the model. Multiple imputations will be used to make the MAR assumption more plausible, via imputation of missing outcomes conditional on additional informative pre- and post-randomisation variables. A mean score approach will also be used to explore the sensitivity of the results of the primary analysis to various systematic departures from MAR [24].

Continuous secondary outcomes (URAM, MHQ, SANE and active/passive range of movement) will be analysed using similar covariance pattern models as used for the primary analysis. Categorical outcomes (recurrence, complications, further procedures and overall hand assessment) will be analysed by appropriate logistic regression models. Time elapsed between the initial treatment of the reference digit and receipt of further treatment to the reference digit will be analysed using a Cox proportional hazards regression model. Secondary analyses will focus primarily on interval estimation, as opposed to hypothesis testing, under either a non-inferiority or superiority testing framework.

#### Economic analysis

The economic evaluation will estimate the relative cost-effectiveness of collagenase compared with surgical treatment.

The short-term effect of collagenase and limited fasciectomy treatments will be assessed using a within-trial cost-utility analysis (CUA) with a 1-year time horizon. If collagenase is cost-effective in the short term, we will extrapolate cost-effectiveness estimates over time. Hence, the primary analysis will be a recurrence model based on DISC evidence. The outcomes of the model will be used to estimate the incremental cost-effectiveness ratio (ICER) of the two treatment options.

Needle fasciotomy is also used as standard care in the NHS for the treatment of Dupuytren’s disease. Since the start of the trial, a number of literature review searches have been conducted to identify publications relevant in this area. Based on the evidence identified through this review, we will conduct a scenario analysis to assess the impact of incorporating needle fasciotomy into the comparison. Finally, a threshold analysis will be used to determine the threshold value of the effectiveness of limited fasciotomy and percutaneous needle fasciotomy, leading to these interventions being cost-effective. The analysis will be consistent with the NICE Guide to the Methods of Technological Appraisal [25] and Decision Modelling for Health Economic Evaluation [26].

Data on resource use and health-related quality-of-life will be collected alongside the trial. Intervention costs will be estimated according to injection and manipulation resource use. Surgical costs will be based on theatre and staff time, consumables and devices, and nights in the hospital after the procedure. The impact of the two interventions in the following costs will be collected using patients’ questionnaires and hospital forms (i.e. outpatient visits, hospital readmissions, A&E visits, general practice, and community and personal health services). The primary analysis will follow an NHS and Personal Social Services (PSS) perspective consistent with that used by the NICE. A secondary analysis will also consider productivity costs and private expenditures.

Health outcomes will be expressed in terms of quality-adjusted life year (QALY) using the EQ-5D-5L (i.e. collected at baseline; 2 and 6 weeks; 3, 6, 12 and 24 months). The EQ-5D-5L health states will be valued following the NICE position statement [27], and utility scores will be converted into QALYs using the area under the curve analysis [28].

Costs and QALY data will be synthesised to generate an incremental cost-effectiveness ratio (ICER). The probability that each intervention is cost-effective will be reported at the cost-effectiveness thresholds of £20,0000 to £30,000/QALY [25] and also £13,000/QALY [29, 30]. Regression methods will be used to allow for differences in prognostic variables. The pattern of missing data will be analysed and handled by multiple imputations if deemed appropriate [31]. Sensitivity analyses were conducted to test the robustness of the results.

#### Interim analyses {21b}

There are no planned interim analyses for the trial or stopping guidelines. An internal pilot study will be conducted to check the initial assumptions about recruitment and feasibility of the trial. Data arising from the pilot phase will be reviewed by the Data Monitoring Committee (DMC) and Trial Steering Committee (TSC) who will recommend if any changes are required and if the trial should continue or not.

#### Qualitative sub-study analysis

Data will be analysed thematically following the conventions established by Braun and Clarke [32]. Interviews will be coded independently with a second-team member ensuring consistency and validity of the coding process. Data analysis will commence before data collection is completed, and coded interviews will be reviewed to inform on-going data collection strategies. Key ideas and themes which help to organise the data will be identified and modelled as part of this process.

The theory will be developed systematically using the constant comparison method [33] in order to maximise consistency and plausibility. Ideas and themes will be reviewed by the broader DISC team and DISC patient and public partners. Emergent theoretical categories and their properties will be contrasted with feedback received from the study’s stakeholders (e.g. principal investigators and members of the study’s patient and public involvement group).

Data from each treatment arm may be considered separately with distinct models for the two study treatments constructed along with a further model which considers Dupuytren’s symptoms and impact. These models will be considered alongside the clinical and economic data to inform the study findings and to inform recommendations for future clinical practice.

#### Photography sub-study analysis

Joint measurements obtained using participant taken photographs will be compared with goniometer readings and measurements obtained using clinician taken photographs, to assess whether there is reasonable agreement between these. Angles will be measured on photographs by observers, using anonymised digital images and a standardised measurement protocol.

Agreement between baseline goniometric measurements and measurements obtained using baseline participant photographs will be assessed for the MCP and PIP joints of the reference digit. The 95% limits of agreement, between the mean of the three repeated goniometric measurements obtained at baseline and the measurement obtained using the participant photograph, will be calculated for each joint following the methods of Bland and Altman [34]. The limits of agreement (and the 95% confidence intervals of these limits) for each joint (MCP and PIP) will be compared with a pre-specified magnitude of clinically acceptable disagreement of 10°.

Secondary analyses will look at the agreement between:

- Goniometric measurements and measurements obtained using patient photographs at 3 and 6 months post-treatment
- Goniometric measurements and measurements obtained using clinician photographs at baseline and 3 and 6 months post-treatment

Limits of agreement values and 95% confidence intervals will be reported for these comparisons by joint and where feasible by digit. Potential predictors of agreement (for both the primary and secondary comparisons) will be explored by regression analysis. Potential predictors include digit measured, contracture severity and image quality.

#### Studies with a trial (SWATs) analysis

Analyses of the retention thank you card SWAT will be conducted using logistic regression, adjusting for SWAT allocation, DISC trial allocation and relevant baseline covariates (if applicable). An unadjusted analysis will also be completed.

Analyses of the retention Christmas card SWAT will be undertaken centrally by the MRC funded PROMETHEUS team.

#### Plans to give access to the full protocol, participant level-data and statistical code {31c}

This document constitutes the full protocol. Following completion of the trial, datasets and statistical code used in this study will be available from the corresponding author on reasonable request.

#### Oversight and monitoring

##### Composition of the coordinating centre and trial steering committee {5d}

YTU will oversee and coordinate the management and running of the study. This will include trial management and coordination, statistical, economic and data management staff.

The trial management group (TMG) will meet quarterly and will consist of the Chief and co-investigators, members of YTU and the Academic Team of Musculoskeletal Surgery (AToMS) responsible for the study.

The Trial Steering Committee (TSC) will comprise two independent members (one clinician and one methodologist) and a patient and public representative. The TSC will provide overall supervision for the trial on behalf of the Sponsor and Funder.

##### Composition of the data monitoring committee, its role and reporting structure {21a}

A Data Monitoring and Ethics Committee (DMEC) will comprise three independent members (two clinicians, one statistician) and a patient and public representative. The DMC will be the only body to have access to unblinded comparative data during the trial should this be required. The DMC will monitor data and make any recommendations to the TSC on whether there are any ethical or safety reasons why the trial should not continue.

##### Adverse event reporting and harms {22}

Adverse events (AEs) related to the affected digit or hand, or to the study intervention or control will be recorded irrespective of whether they are expected or unexpected. AEs that may be expected are detailed in the Summary of Product Characteristics (for the intervention) [4] and in Table 3 for the control (See the “Outcomes” section). Any event which has a reasonable, suspected causal relationship to the study medication (intervention) will be deemed an adverse reaction.

Serious adverse events that are deemed related to the research and are unexpected will be reported to the Research Ethics Committee (REC) who approved the study. All AEs will be routinely reported to the TMG, TSC, DMEC and Sponsor. The DMEC will be responsible for reviewing related and unexpected serious adverse events.

Adverse events will be collected and reported through review of any complications associated with the intervention and control at each study visit and via spontaneous report should the participant report any adverse events between study visits. All AEs that are related to the study medication or procedures and which are unresolved at initial reporting will be followed up until resolution or the event is considered to be stable.

All adverse events will be reported in any associated, relevant publications arising from this trial.

##### Frequency and plans for auditing trial conduct {23}

The study will be conducted in accordance with the currently approved protocol, ICH Good Clinical Practice (GCP), relevant regulations, standard operating and trial-specific procedures.

Regular monitoring will be performed according to ICH GCP and the DISC Monitoring Plan. Data will be evaluated for protocol compliance and accuracy in relation to source documents. Following written standard operating and trial-specific procedures, the monitors will verify that the clinical trial is conducted and data are generated, documented and reported in compliance with the protocol, ICH GCP and the applicable regulatory requirements.

##### Plans for communicating important protocol amendments to relevant parties (e.g. trial participants, ethical committees) {25}

Important protocol modifications will be submitted to the REC and Health Research Authority (HRA) for approval having been agreed with the Funding Body, Sponsor, TSC, DMEC and the TMG. Minor modifications to the protocol will be agreed upon with the TMG and Sponsor before submission for approval to the REC and HRA. All amendments will be implemented in accordance with the guidance of the HRA. Trial participants will be written to, if necessary, to explain any changes.

##### Dissemination plans {31a}

Results from this study will be written up and submitted to peer-reviewed journals. A publications policy will be generated in advance to detail authorship, acknowledgements and review processes for any publications arising from the DISC Trial. Authorship will be determined in accordance with the International Committee of Medical Journal Editors.

## Discussion

The DISC trial has been designed to provide a robust answer to guide the treatment of Dupuytren’s contracture as regards the effectiveness of collagenase, in terms of patient-reported hand pain and functionality as assessed by the PEM. Overall, collagenase would have to not be inferior to limited fasciectomy surgery in order for it to be recommended for widespread use, and the DISC Trial will enable this comparison to be made. The study will assess rates of recurrence between the two treatments which is an important consideration for both patients and healthcare professionals when discussing available treatment options. Key also to patients, and the wider economy, is the return to function as soon as possible following treatment, which is also assessed within the DISC trial.

As noted, collagenase was withdrawn from the UK and European market for commercial reasons as of March 2020. The DISC trial team worked to secure sufficient stocks to continue recruitment to the contracted target. These efforts were driven by a clear steer by clinicians (site principal investigators and co-applicants) that results of this study continue to have an important bearing on treatment options offered to patients. These efforts were reviewed and supported by the funder (NIHR) and DISC trial oversight committees.

Collagenase continues to be produced and marketed in the USA for treatment of Dupuytren’s contracture. Therefore, this study would provide reliable information on which to base the reintroduction for clinical use in Europe. A similar study cannot be commissioned in the UK or Europe, given the current market withdrawal, making the DISC trial the only opportunity for a study in this region to collect reliable data in relation to the effectiveness of collagenase compared with limited fasciectomy. The results also provide a key component to a comprehensive assessment of treatment options for Dupuytren’s contracture. A plan of research for this is already underway with funding secured as part of the UK National Institute for Health Research-funded HAND2 trial [NIHR: 127393; ISRCTN: 18254597], which includes a package of work to bring together all treatment comparisons for Dupuytren’s contracture in an individual patient data network meta-analysis.

### Trial status

Recruitment to the DISC trial began in June 2017 and will complete by the 30th of September 2021.

## Supplementary Information

**Additional File 1.** DISC Trial Participating Sites.

## Data Availability

This document constitutes the full protocol. Following completion of the trial datasets and statistical code used in this study will be available from the corresponding author on reasonable request.

## Abbreviations

AE: Adverse events
BSSH: British Society for Surgery of the Hand
CI: Confidence interval
CONSORT: Consolidated Standards of Reporting Trials
CUA: Cost utility analysis
DIP: Distal interphalangeal joint
DISC: Dupuytren’s interventions surgery vs collagenase trial
DMC: Data monitoring committee
EQ-5D-5L: EuroQoL 5 domain 5 level questionnaire
GP: General practitioner
HRA: Health research authority
ICER: Incremental cost-effectiveness ratio
ICH GCP: International Conference on Harmonisation Good Clinical Practice
ID: Identification
ISRCTN: International Standard Randomised Controlled Trial Number
MAR: Missing at random
MCP: Metacarpophalangeal joint
MHQ: Michigan Hand Questionnaire
NHS: National Health Service
NICE: National Institute for Health and Care Excellence
QALY: Quality-adjusted life year
PEM: Patient evaluation measure
PIP: Proximal interphalangeal *joint*
*PSS*: *Personal social services*
*REC*: *Research ethics committee*
RTT: Referral to treatment time
SANE: Single Assessment Numeric Evaluation Measure
SWAT: Study within a trial
TSC: Trial steering committee
TMG: Trial management group
UK: United Kingdom
UKCRC: United Kingdom Clinical Research Collaboration
URAM: Unité Rhumatologique des Affections de la Main Scale
USA: United States of America
