# Supplementary material for: Dupuytren’s interventions surgery versus collagenase (DISC) trial: study protocol for a pragmatic, two-arm parallel-group, non-inferiority randomised controlled trial
Source: Trials. 2021 Sep 30;22:671. doi: 10.1186/s13063-021-05595-w (PMC8481756; doi:10.1186/s13063-021-05595-w)
Supplement: Supplementary file 1 — Additional File 1. DISC Trial Participating Sites. [file 13063_2021_5595_MOESM1_ESM.docx]

Supplementary File 1 - DISC Trial Participating Sites

| Basingstoke and North Hampshire Hospital | Bedford Hospital |
| --- | --- |
| Royal Blackburn Hospital | Chelsea and Westminster Hospital |
| City Hospitals Sunderland | Derriford Hospital (Plymouth) |
| Glenfield Hospital (Leicester) | Gloucestershire Royal Hospital |
| James Cook University Hospital (South Tees) | King’s College Hospital |
| Lister Hospital | Northern General Hospital (Sheffield) |
| Princes Royal Hospital (Brighton) | Queen Alexandra Hospital (Portsmouth) |
| Robert Jones and Agnes Hunt Orthopaedic Hospital (Oswestry) | Royal Bolton Hospital |
| Royal Cornwall Hospital | Royal Derby Hospital |
| Royal Liverpool and Broadgreen Hospital | Royal Orthopaedic Hospital (Birmingham) |
| Royal Victoria Infirmary (Newcastle) | Russell’s Hall Hospital (Dudley) |
| Southampton General Hospital | St Georges Hospital (London) |
| University Hospital of Coventry and Warwickshire | University Hospital Monklands |
| University College Hospital (London) | University Hospital of North Tees |
| Warrington and Halton Hospital | Western General Hospital (Edinburgh) |
| Wrightington Hospital |  |
